# Smoking, female gender and PI use are associated with decreasing renal function in TDF-containing HAART

**DOI:** 10.1186/1758-2652-13-S4-P84

**Published:** 2010-11-08

**Authors:** A Uglietti, C Gervasoni, E Gabrielli, S Di Giambenedetto, R Cauda, R Esposito, P Grima, G Di Perri, R Maserati, M Galli

**Affiliations:** 1 Foundation IRCCS Policlinico San Matteo Hospital, Infectious Diseases, Pavia, Italy; 2 L. Sacco Hospital, Department of Clinical Sciences, Milano, Italy; 3 Policlinico Gemelli, Infectious Diseases, Roma, Italy; 4 Policlinico Universitario, Infectious Diseases, Modena, Italy; 5 Santa Caterina Novella Hospital, Infectious Diseases, Galatina, Italy; 6 Amedeo di Savoia Hospital, Infectious Diseases, Torino, Italy; 7 Foundation IRCCS Policlinico San Matteo, Infectious Diseases, Via Taramelli 5, Pavia, Italy; 8 L.Sacco Hospital, Department of Clinical Sciences, Milano, Italy

## Purpose of the study

Nephrotoxicity of tenofovir (TDF)-containing HAART may be associated to several factors related to treatment or to patient's characteristics. We tried to assess Glomerular Filtration Rate (GFR) changes and their relationship with multiple clinical parameters in an Italian cohort receiving a TDF-containing HAART.

## Methods

OSMA-1 (Observational Study on Metabolic Abnormalities), a multicenter Italian study, was designed to evaluate since February 2008 the efficacy and the safety of TDF-based regimen in a real-life clinical setting. HIV infected, therapy naïve subjects were enrolled. GFR was estimated using Cockcroft-Gault (CG), Modification of Diet in Renal Disease (MDRD) and Chronic Kidney Disease Epidemiology Collaboration (CKD-EPI) equations. Statistical analyses used a parametric test and a mixed model was used to analysis changes from baseline to 6, 12, and 24 months, with the different variables as fixed effect plus visit (categorical) and baseline value (continuous as covariate).

## Summary of results

We consecutively enrolled 172 patients (91.3% Caucasian; 72.2% males; mean age 39.3 yrs; 48.8% heterosexual; 36.6% smokers). At baseline median CD4+ cell count was 225 cells/µL (range 2 - 701), HIVRNA > 100000 copies/mL in 43.6%. Median Body Mass Index (BMI) was 22.6 for males and 21.9 for females. A boosted PI was given in 60.5% of cases. At 6-mos evaluation, Women had greater declines in GFR vs. Men, independently from the GFR equation used (means in W vs. M for CG, MDRD and CK-EPI, respectively: -7.2; -12.1; -10 vs. 0.5; -1.3; -0.3 mL/min/1.73 m2, p=0.0001 to 0.0026). No further decline was documented after 6 months. Factors associated with demography or treatment are shown in Table 1.

**Table 1:** 

Variable	CG	p-value	MDRD	p-value	CK-EPI	p-value
Increasing Age	decrease	0.0001	decrease	0.043	decrease	0.0006

Increasing BMI	increase	<0.0001	NA	ns	decrease	0.059

PI/r usage	NA	ns	decrease	0.072	decrease	0.023

Smoking	decrease	0.073	decrease	0.012	decrease	0.05

Female sex	decrease	0.0001	decrease	0.0009	decrease	0.0026

## Conclusions

Our data suggest that long-term use of TDF is associated with a modest decline in GFR that occurs predominantly at the beginning (in the first 6 months) of therapy in HAART-naive women. However, the decline is small, does not seem to worsen over time, and may not have a relevant clinical effect. A decreasing GFR was also found in elder patients, in smokers and when a boosted PI is used.

